# Current Neonatal Resuscitation Practices among Paediatricians in Gujarat, India

**DOI:** 10.1155/2014/676374

**Published:** 2014-02-12

**Authors:** Satvik C. Bansal, Archana S. Nimbalkar, Dipen V. Patel, Ankur R. Sethi, Ajay G. Phatak, Somashekhar M. Nimbalkar

**Affiliations:** ^1^Department of Paediatrics, Pramukhswami Medical College, Karamsad, Anand, Gujarat 388325, India; ^2^Department of Physiology, Pramukhswami Medical College, Karamsad, Anand, Gujarat 388325, India; ^3^Central Research Services, Charutar Arogya Mandal, Karamsad, Anand, Gujarat 388325, India

## Abstract

*Aim*. We assessed neonatal resuscitation practices among paediatricians in Gujarat. *Methods*. Cross-sectional survey of 23 questions based on guidelines of Neonatal Resuscitation Program (NRP) and Navjaat Shishu Suraksha Karyakram (NSSK) was conducted using web-based tool. Questionnaire was developed and consensually validated by three neonatologists. *Results*. Total of 142 (21.2%) of 669 paediatricians of Gujarat, India, whose e-mail addresses were available, attempted the survey and, from them, 126 were eligible. Of these, 74 (58.7%) were trained in neonatal resuscitation. Neonatal Intensive Care Unit with mechanical ventilation facilities was available for 54% of respondents. Eighty-eight (69.8%) reported correct knowledge and practice regarding effective bag and mask ventilation (BMV) and chest compressions. Knowledge and practice about continuous positive airway pressure use in delivery room were reported in 18.3% and 30.2% reported use of room air for BMV during resuscitation. Suctioning oral cavity before delivery in meconium stained liquor was reported by 27.8% and 38.1% cut the cord after a minute of birth. Paediatricians with NRP training used appropriate method of tracheal suction in cases of nonvigorous newborns than those who were not trained. *Conclusions*. Contemporary knowledge about neonatal resuscitative practices in paediatricians is lacking and requires improvement. Web-based tools provided low response in this survey.

## 1. Introduction

The life of a foetus in utero and the independent existence of a newborn are two vastly varied conditions requiring complex transitions. Birth asphyxia contributes to 19% of the 4 million neonatal deaths worldwide every year. In addition to its contribution to mortality, birth asphyxia can result in cognitive impairment, epilepsy, cerebral palsy, and chronic diseases in later life [1]. These numbers assume significance in Indian settings where neonatal mortality rate of 33 contributes to about 75% of the infant mortality rate of 47 as figures from 2010 reveal. This contribution of neonatal mortality to infant mortality has been increasing over the past decade as measures to reduce infant mortality are becoming effective [2].

Approximately 10% of newborns (4–7 million per year) require some form of assistance at birth. This makes neonatal resuscitation a frequently performed medical intervention [3–5]. As per the updated (October 2010) recommendations of International Liaison Committee on Resuscitation (ILCOR), Neonatal Resuscitation Program (NRP) of American Heart Association (AHA) and American Academy of Paediatrics (AAP), at least one trained person is required to be present during delivery [4]. This requires that the healthcare personnel involved need to be abreast with the latest recommendations and should follow them in their clinical practice. The Indian Academy of Pediatrics (IAP) and National Neonatology Forum (NNF) of India currently follow NRP guidelines. IAP in collaboration with National Rural Health Mission of Government of India developed Basic Newborn Care and Resuscitation Programme (BNCRP) of Navjaat Shishu Suraksha Karyakram (NSSK) adopted from NRP guidelines for grass root workers as well as paediatricians [6].

A questionnaire based survey from Haryana, India, showed poor knowledge and practices of neonatal resuscitation among the healthcare personnel attending deliveries [7].

There is lack of information regarding neonatal resuscitation practices prevalent among paediatricians of Gujarat. This study assesses this issue with the help of web-based tool.

## 2. Materials and Methods

### 2.1. Setting

This survey was conducted amongst paediatricians within the state of Gujarat over a period of 4 months from April to July 2012. The study was approved by the Human Research Ethics Committee of HM Patel Centre for Medical Care and Education, Karamsad.

### 2.2. Data Collection

**Figure 1:**
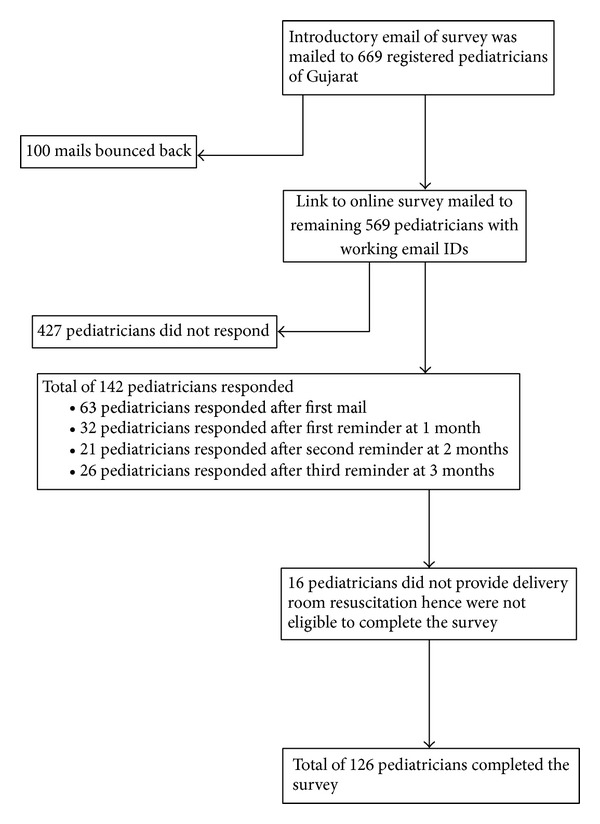
Recruitment process.

The questionnaire was based on revised 2010 NRP guidelines as well as NSSK guidelines and was developed, pilot-tested, and consensually validated by SMN, DVP, and ASN. It consisted of 23 multiple-choice clinical knowledge based questions, and responses were based on common interventions performed during neonatal resuscitation (the Appendix). The questionnaire was placed on an online survey website, https://www.surveymonkey.com/. The recruitment process is summarized in Figure 1. The access to the data collected over the server was password-protected. Due care was taken to prevent data loss or data entry error. The paediatricians who did not provide delivery room resuscitation in their setup were excluded.

Data were downloaded as MS Excel 2010 spreadsheets and analysed using SPSS (version 14). Univariate analysis was done to compare the practices between trained and untrained care givers. A *P* value less than 0.05 was considered significant.

## 3. Results

Out of 1,169 registered paediatricians in the state of Gujarat, e-mail addresses of 669 paediatricians were available. Over the span of 4 months, 142 (24.9%) paediatricians responded from 569 working email addresses and from them 126 were eligible for the survey (Figure 1).

Out of 126 paediatricians, 68 (54%) were associated with Neonatal Intensive Care Unit (NICU) with mechanical ventilation facility, 84 (66.7%) performed more than 20 resuscitation, and 67 (53.2%) attended more than 100 deliveries in the last one year. Only 73 (57.9%) reported to conduct resuscitation of high risk/unstable infants in the new-born corner in the delivery room under radiant warmer. Most of the participants 93 (73.8%) reported having saturation monitor in the delivery room, but only 34 (27%) reported availability of oxygen blender. Although recommended, only 23 (18.3%) reported using continuous positive airway pressure (CPAP) in the delivery room. Forty-six (36.5%) of the paediatricians had NSSK training, while 55 (43.7%) were trained in NRP in the last three years. Practice of positive pressure ventilation in delivery room was performed by self-inflating bag flow inflating bag and Neopuff (T piece resuscitator) in 103 (81.7%), 2 (1.5%), and 18 (14.2%) respondents, respectively.

Of 126 paediatricians, 88 (69.8%) reported correct knowledge and practice regarding effective bag and mask ventilation and chest compressions. Only 46 (36.5%) of the paediatricians applied plastic/thermal wraps for extremely low birth weight newborns, which is a recommended practice. Similarly, only 48 (38.1%) participants followed the recommended practice of cutting the umbilical cord after a delay of one minute. Many participants 78 (61.9%), adopted the current recommendations of endotracheal suctioning of nonvigorous newborn in cases of meconium stained liquor. Thirty-five (27.8%) followed oral cavity suctioning before delivery of shoulder.

The participants who underwent NRP training were following correct practices as compared to those without the training with respect to meconium stained liquor (80% versus 53.1%, *P* = 0.002), but no significant difference was found with respect to application of plastic/thermal wraps for extremely low birth weight babies (43.6% versus 34.9%, *P* = 0.33) and timing of cutting of the umbilical cord (45.5% versus 36.1%, *P* = 0.30). The use of bag and mask with room air was not significantly different (84.4% versus 82.4%, *P* = 0.49) between those who underwent NSSK/BNCRP training and those who did not.

## 4. Discussion

This survey on resuscitation practices in Gujarat represents the difference between practices of the individual providers and the latest 2010 NRP guidelines. The results obtained are mostly reflective of the practices followed in advanced neonatal units as the majority (54%) of participants were from NICU with ventilation facility.

There was marked variation amongst the respondents regarding the time of clamping and cutting of umbilical cord; 61.9% of the respondents immediately cut the cord, whereas the rest waited for one minute. Consensus on Science with Treatment Recommendations (CoSTR) recommend delayed clamping of cord in both term and preterm uncomplicated deliveries [3, 8]. This practice is associated with decreased incidence of IVH and higher blood pressures during stabilization and thus improved neonatal outcome.

The practice of the intrapartum suctioning of oropharynx and nasopharynx before the delivery of the shoulder is no longer recommended [9], but 27.8% of respondents in this survey still did it. Majority of the respondents agreed on endotracheal suctioning of nonvigorous babies only, which is recommended. Earlier endotracheal suction of all infants, whether vigorous or nonvigorous, was performed in an effort to decrease the incidence of meconium aspiration syndrome; then, two large randomized controlled trials, questioned this practice [10, 11]. As a result, endotracheal suctioning of vigorous infants with meconium stained amniotic fluid (MSAF) is no longer recommended [8].

The latest NRP guidelines based on few studies [12, 13] recommend the monitoring of saturation of newborns in the delivery room. Pulse oximeter gives a continuous audible heart rate signal in addition to providing oxygen saturations, thereby allowing the resuscitators to concentrate on other tasks. In the delivery room, ideally a pulse oximeter should be used—one with highest sensitivity and lowest average signal detection time. In our survey, 73.8% of the paediatricians had saturation monitors in the delivery room. This information is encouraging for a resource-limited country like India, especially, as a recent survey in UK showed that only 58% of tertiary units and 29% of nontertiary units regularly used pulse oximeters [14].

The latest NRP and ILCOR guidelines recommend the use of room air for initial resuscitation of term infants [3, 4]. This survey shows that 63.5% of the respondents still initiate resuscitation with oxygen. This finding may reflect a gap in knowledge or lack of universal acceptance of NRP guidelines or both. A similar survey conducted in 2012 in UK [14] showed that 84.5% of individuals were using room air whereas 90% of the participants from level-three units in Canada [15] were following the same. This shows an earlier adaptation of the newer guidelines, although room air has been incorporated in the guidelines since 2005 in Canada. In an earlier survey in 2004 from Australia and New Zealand, most healthcare personnel utilized oxygen as per the guidelines prevalent during those times [5]. Thus, there is a better adherence to guidelines in the developed world. In our survey, only 27% of paediatricians had oxygen blenders in the delivery room. In contrast, 97% of neonatologists working in tertiary care settings in Canada [15] and 71.7% participants in UK [16] were using oxygen blenders. This shortcoming though unacceptable, is a reality in a resource-limited nation like India. Appropriate emphasis must be endowed to ensure availability of basic infrastructural requirements for high quality resuscitation.

The temperature of all newborns should be maintained at 37.0 ± 0.5°C [17]. In very low birth weight infants there is greater incidence of heat loss and about 25% have temperature <35°C at the time of admission [18]. This hypothermia gravely affects the prognosis of the newborns [19]. To prevent insensible heat loss, wrapping of high-risk infants is recommended [3, 8, 20]. The EPICure study showed that hypothermia (temperature < 35°C) was associated with increased mortality rates in extremely low birth weight (ELBW) newborns [19]. These led to two prospective randomized trials that reported the benefit of polythene wraps for preventing heat loss amongst ELBW infants [21, 22]. The infant's head was dried and the polythene wrap was covered over the body without drying. This direct application reduces evaporative and convective heat losses [23]. In this survey, 36.5% of the respondents used plastic/thermal wraps. Lack of awareness, financial constraints, and unavailability of proper sterilization facilities appear to hinder its global acceptance.

The latest NRP algorithm and ILCOR recommend the use of CPAP in delivery room. Many animal studies have demonstrated the utility of peak end expiratory pressure (PEEP) in maintaining functional residual capacity and surfactant function and reducing lung injury [24–26]. In this survey, we found only 18.3% of paediatricians using delivery room CPAP. However, there was no significant difference noted in the practice by those who have attended any neonatal resuscitation training program in the past three years and those who have not, probably reflecting infrastructural and financial constraints. But, it has also been shown previously that the knowledge gained by participating in such training courses is high but is only partially retained [27]. Hence, this noncompliance can be attributed to both of these factors.

There is a need to follow up the process of knowledge and skills gained by the trainees into clinical practice, by periodical refresher courses and evaluations. This would lead to baseline improvement in competence by adherence to recommended resuscitation guidelines and thereby improve quality of care provided to newborns immediately after birth [28, 29]. The respondents were accustomed to basic resuscitative practices, but there were undeniably certain grey areas, where awareness needs to be increased. There were a total of 5 NRP trainings conducted in the years 2011 to July 2012 involving 40 participants in each training programme. From these 200 trained participants, only 28 were paediatricians and the rest were resident doctors, in paediatrics, MBBS doctors and nurses. From 559 persons trained in NSSK during the same period, only 73 were paediatricians. As there is no legal requirement by the regulatory authorities to complete NRP/NSSK before attending deliveries, it is expected that this gap in knowledge will continue. Innovative methodologies in training and flexible courses need to be devised so that new knowledge reaches those who can use it the most. Varied adoption of practises followed by trained paediatricians in this study can be explained by theory of Diffusion of Innovations of Everett Rogers [30]. However, we did not evaluate the causes of failure of adoption of the current practices of neonatal resuscitation.

There was no difference in the practice like cutting the umbilical cord, applying plastic/thermal wraps or utilizing BMV between trained and nontrained paediatricians. Studies on neonatal resuscitation practices have been conducted in various countries. In Canada, a clear gap in recommendations and practices was observed. It was also found that certification in NRP did not ensure competency and compliance with established standards of care [31]. Similar gaps have been reported in studies done in Muscat, Poland, Spain, Nepal, and United Kingdom [16, 32–35].

We observed a low response rate for the survey and this may be a threat to the generalizability of surveys conducted by e-mail or through internet-based modalities. The low response rate in this study is in contrast with the higher response rates reported in Canada (55%) and Australia (64%) which utilized the similar methodology [15, 36].

This web-linked survey method merged the process of data collection and data entry allowing the investigators to proceed with analysing the data. It has a greater reach and an option of real time monitoring. There are also less chances of data loss. More experience with such web-linked surveys is needed to establish their overall effectiveness. In this survey, we did not differentiate between paediatrician and neonatologist. There is an issue of compliance in web-linked method of surveys and it is more complex than traditional methods. In this survey, we did not include the question pertaining to total duration of practice in paediatrics.

## 5. Conclusions

This survey has identified areas of nonuniformity and lack of awareness amongst paediatricians for practices followed for neonatal resuscitation. There are evident gaps in the knowledge and compliance for the latest NRP and NSSK norms amongst the paediatricians of Gujarat. Research into effective dissemination of these guidelines is imperative. The web-based survey though reported low response rate had greater reach.

*What Is Already Known on This Topic*. Resuscitation at birth has a major role in improving morbidity and mortality of neonates. The guidelines are repeatedly revised; last revision in NRP based on ILCOR is done during 2010. Updating the practice needs to be done to improve birth outcomes. 

*What This Paper Adds*. The contemporary knowledge of current neonatal resuscitation guidelines is low even in trained paediatricians in Gujarat. Research into effective dissemination of guidelines is imperative.

## Appendix

### Web-Based Questionnaire

Name:

Institution:

If you do not provide delivery room resuscitation in your setup, please go to last question and return the form

1. Please indicate the level of NICU in your center
  - Stabilization of newborn babies and referral
  - Admission of LBW babies for sepsis, jaundice, exchange transfusion, feeding problems, and so forth
  - Facilities available for ventilation
2. Number of neonates resuscitated by you in the last year
  - 1-2
  - 3–7
  - 8–16
  - More than 20
3. Number of deliveries (vaginal or LSCS) attended in the last year
  - Less than 10
  - 10–50
  - 50–100
  - More than 100
4. Where do you resuscitate high-risk/unstable infants after delivery?
  - In the dedicated newborn corner in the delivery room
  - In a separate room near the delivery room
  - In the NICU or separate adjacent room
  - Anywhere
5. Device of your choice when providing positive pressure ventilation with a mask in the delivery room
  - Self-inflating resuscitation bag
  - Anaesthesia bag
  - Neopuff T-piece resuscitator
  - Other
6. How do you begin ventilation of the term neonate with bag and mask during resuscitation?
  - Oxygen attached to bag and mask but without reservoir
  - Oxygen attached to bag and mask with reservoir
  - Only bag and mask without any reservoir or oxygen
  - Neopuff
7. At what rate do you give breaths by bag and mask while resuscitating term neonates?
  - 20–30/min
  - 20–30 in 90 sec
  - 20–30 in 30 sec
  - 20–30 in 15 sec
8. Do you have a saturation monitor in the resuscitation area of delivery room?
  - Yes
  - No
9. Do you have an oxygen blender in the resuscitation area of delivery room?
  - Yes
  - No
10. Do you use CPAP or PEEP in the delivery room?
  - Yes
  - No
11. If you use CPAP/PEEP in the delivery room, what level of pressure do you use?
  - 4
  - 5
  - 6
  - 7
12. Which of the following is true about chest compressions?
  - Area is below the xiphoid process of sternum
  - Area is above the nipple line
  - Done at compression: ventilation ratio of 1 : 2
  - Using palm for compression
  - Using thumbs for compression
13. Free flow oxygen can be given reliably by a mask attached to self-inflating bag
  - True
  - False
14. For persistent apnea, just after birth, what would you do?
  - Continue tactile stimulation a little bit more vigorously
  - Give positive pressure ventilation promptly
  - Give free flow oxygen
15. The best indicator of effective bag and mask ventilation is
  - Rising heart rate and audible breath sounds
  - Rise in oxygen saturation
  - Chest movements
  - None of the above
16. During chest compression how much pressure do you use?
  - Depress the sternum to 1/3rd of AP diameter of chest
  - Depress the sternum to 1/2 of AP diameter of chest
  - There is no strict guideline; it varies depending upon the weight of the baby
  - Go on increasing pressure till there is no response
17. Have you undergone the NSSK/BNCNRP program of the IAP in the last three years?
  - Yes
  - No
18. Have you undergone the NRP program of the NNF in the last three years?
  - Yes
  - No
19. How long do you resuscitate a neonate who has asystole and not improving with all measure?
  - 5 minutes
  - 10 minutes
  - 15 minutes
  - 20 minutes
20. For term babies born through meconium stained liquor, one of the following is to be done
  - Suction of oral cavity before delivery of shoulder
  - Endotracheal suction of active baby (vigorous)
  - Endotracheal suction of nonvigorous baby
  - Endotracheal suction of all babies born through meconium stained liquor
21. Do you routinely apply plastic/thermal wraps for extremely low birth weight (ELBW) babies immediately after birth?
  - Yes
  - No
22. What is the routine practice in your delivery room regarding cutting of the umbilical cord?
  - Cord is cut immediately after the delivery of the baby
  - Cord is cut after a delay of a minute of the delivery of the baby
  - Cord is cut after pulsations stop
  - Cord is cut after 5 minutes
23. After vaginal delivery, the baby is placed at the following place in your primary area of practice
  - Under the radiant warmer in newborn care corner
  - On the side of the mother
  - On the chest/abdomen of the mother.

## Abbreviations

AAP: American Academy of Paediatrics
AHA: American Heart Association
BMV: Bag-mask ventilation
BNCRP: Basic Neonatal Care Resuscitation Program
CPAP: Continuous positive airway pressure
HREC: Human Research and Ethics Committee
ILCOR: International Liaison Committee on Resuscitation
LBW: Low birth weight
LSCS: Lower segment caesarean section
NICU: Neonatal Intensive Care Unit
NNF: National Neonatology Forum
NRP: Neonatal Resuscitation Program
NSSK: Navjaat Shishu Suraksha Karyakram
PEEP: Peak end expiratory pressure
USA: United States of America.
